# Antipsychotic patterns in outpatients with schizophrenia in China: A cross sectional study

**DOI:** 10.1097/MD.0000000000026912

**Published:** 2021-08-13

**Authors:** Xiaobing Guo, Hongye Yu, Hu Wang

**Affiliations:** a The National Clinical Research Center for Mental Disorders & Beijing Key Laboratory of Mental Disorders, Beijing Anding Hospital, China; b Advanced Innovation Center for Human Brain Protection, Capital Medical University, Beijing, China.

**Keywords:** antipsychotics, China, long-acting injectable antipsychotic, patterns of use, schizophrenia

## Abstract

It is essential to monitor pharmacological treatment for schizophrenic outpatients regularly in clinical practice. Especially in China, the situation of common prescribing patterns remains unclear. The objective of this study is to reveal real-world treatment prescription patterns of antipsychotics for schizophrenia patients in a representative large tertiary hospital in China.

This study is a cross-sectional observational analysis of outpatients with schizophrenia in a large tertiary psychiatric hospital in Beijing, China, from May 11th to 24th, 2019. Data on subjects’ socio-demographic and clinical characteristics, prescriptions of psychotropic drugs were collected from the electronic medical record (EMR) system with a standardized protocol. A multivariate analysis was performed to explore the potential association between antipsychotics treatments and subjects’ characteristics.

Of the 1940 patients included in this study, only 1470 (75.77%) patients were prescribed antipsychotic medications. 1228 (83.53%) patients were prescribed second-generation antipsychotics (SGAs), 202 (13.74%) patients were treated only with first-generation antipsychotics (FGAs), 40 (2.72%) were prescribed both SGAs and FGAs. The proportion of single SGAs prescriptions was significantly higher than that of single FGAs antipsychotics in each course of monotherapy group, especially among patients with the course less than 2 years (96.08%). Risperidone was most frequently prescribed antipsychotic medication during the study (29.86%, 439 out of 1470). Intermediate-acting sedative benzodiazepines were the most commonly co-prescribed psychotropic class at 23.66%. Long-acting injectable antipsychotics (LAIs) could be the prescribing trend in clinics. Disease course, self-paying cost and LAI antipsychotic use were independently associated with antipsychotics treatments.

Second-generation antipsychotics showed domination in prescriptions. More concerns should be paid with concomitant psychiatric medications in clinics.

## 1 Introduction

Schizophrenia is a complex, chronic psychiatric disorder characterized by active and superimposed psychosis, which principally involves delusions, hallucinations, and disorganized thoughts, as well as cognitive deficits and social dysfunction.^[1]^ It is affecting around 21 million people with the lifetime prevalence of schizophrenia arounds 0.30% to 0.66% worldwide,^[2,3]^ however a reported prevalence of schizophrenia in China is 0.7% in 2019.^[4]^ This indicates that there are more large amounts of schizophrenia patients in this country. This debilitating disorder is associated with reduced quality of life and shortened life expectancy. Consequently, the ultimate objectives of treatments are to improve functional capabilities, minimize residual symptoms and reduce the chance and time of relapse.^[5]^

Antipsychotic drugs are the cornerstone of the treatment, which is often lifelong. Both the first- (FGAs) and the second-generation antipsychotics (SGAs) have general D2 receptor antagonistic pharmacodynamic properties in the brain.^[6]^ Three dimensions are commonly considered based on the recommendations of treatments:

1. the use of FGAs and/or SGAs;
2. receiving antipsychotic monotherapy and/or and polypharmacy, and
3. optimal dosage form of antipsychotics (oral and/or long-acting injectable [LAIs]).

The SGAs are thought to be more efficacious in the treatment of negative symptoms and provide less association with extrapyramidal symptoms compared with the FGAs and are recommended by most guidelines as first-line therapy for schizophrenia.^[7]^ In spite of that, the implementation of clinical practice guidelines in real world has proven challenging to achieve,^[8]^ reflected by vast varieties in the prescription patterns of antipsychotics between different comparable regions and countries.

Several commonly prescribed SGAs in China include risperidone, olanzapine, clozapine, aripiprazole and paliperidone.^[9,10]^ Surveys of the patterns of antipsychotic prescription in the Beijing Anding Hospital affiliated with the Capital Medical University, as one of the largest tertiary psychiatric hospitals in China, are prospective in China but with the scarcity of published data on the prevalent use of these drugs. Meanwhile, with the promotion and implementation of the digital development of healthcare informatization in China, the electronic medical record (EMR) system provides richer resources for real-world studies. The study aims to reveal the use of antipsychotics in real-life settings, by conducting an evaluation of the 2-week prescription patterns of antipsychotics in outpatients in a Beijing large tertiary psychiatric hospital, compared with the international recommended guidelines of monotherapy. In addition, we focused on the impacts of demographic or clinical factors on patients’ prescriptions with schizophrenia.

## 2 Methods

### 2.1 Study design

This study belongs to the project “Study on individualized optimal treatment regimen of antipsychotics (SQ2016YFSF110100)”. It was a single-center, cross-sectional observational survey on prescription trends of psychotropic medications conducted between May 11th and 24th, 2019, in Beijing Anding Hospital. The data were collected based on the EMR system of outpatients who were diagnosed with the schizophrenia spectrum disorder. All data were anonymous so the study did not directly contact patients. It also got the waiver of informed consent which given by Ethics Committee of The Anding Hospital Affiliated to Capital Medical University. The study was enrolled in the Chinese Clinical Trial Registry with the registration number of ChiCTR1900025065.

### 2.2 Setting

Patients are divided into 2 groups based on the antipsychotic prescription patterns: the antipsychotic monotherapy group and the polypharmacy group. Monotherapy is defined as having single antipsychotic medication, whereas antipsychotic polypharmacy is identified as taking 2 or more antipsychotics simultaneously. Patients were stratified by course of disease: less than 2 years, 2 to 5 years, 5 to10 years, and more than 10 years. This study also focused on the following major stratifications of patients viz. age groups (<18, 18–65, >65 years), groups of dosage forms, and some different schemes of health insurance. Concomitant prescriptions for these outpatients of other psychiatric drugs are classified as benzodiazepines, antidepressants, mood stabilizers, and antihistamines.

### 2.3 Participants

Patients met the selection criteria for this study:

1. being arrived at the definitive diagnosis as schizophrenia according to the ICD-10 guidelines;
2. being treated as only outpatients;
3. being recorded by the EMR system in Beijing Anding Hospital.

### 2.4 Data source

All data retrieved in the study were extracted from the EMR system developed in Beijing Anding Hospital. In accordance with the Personal Information Protection Law of the People's Republic of China, the EMR data were de-identified and requested anonymity prior to research use. In order to get standardized and consolidate data, the following operations were conducted: a) manual data extraction, b) data inspection, c) construction of analytical tables.

### 2.5 Variables

Relevant demographic and clinical information, including age, gender, chief complaint, course of the disease, history record, and type of psychotropic medications were categorized. Detailed information on medication patterns was classified into antipsychotic monotherapy or polypharmacy of different generations (FGAs/SGAs), and other psychotropic classes been co-prescribed with. The types of health care costs were also collected and segmented into public medical insurance or self-paying subjects.

### 2.6 Statistical methods

The analyzes were performed by using SAS software version 20.0 (SAS Institute Inc, Cary, NC, USA) for Windows. The statistical significance of the two-tailed test was set at *P* < .05. The normality of distribution of continuous variables was assessed with the Kolmogorov-Smirnov test. Measures of central tendency ± standard deviation are shown for quantitative variables. Qualitative variables were presented by frequency and percentage. The Chi-Squared test was used when contrasting categorical variables. A backward stepwise logistic regression analysis was performed to explore the potential association of antipsychotic polypharmacy with the antipsychotics used most often, use of LAIs, SGAs used and other variables in the study. Variables included in the study were gender, age, psychiatric co-medication, course of the disease, antipsychotic used and the use of LAI antipsychotics.

## 3 Results

### 3.1 Patient characteristics

A total of 1940 patients with a diagnosis of schizophrenia were included during the study period. As shown in Table 1, 1940 patients, as valid samples met the study selection criteria. The mean age was 43.30 ± 16.36 years and more than half of the subjects were female (1,042, 53.71%). Among them, 66.24% of patients obtained public medical insurance, and the rest of them were self-paying patients.

**Table 1:** Patient characteristic at baseline.

Variables	N = 1940
Gender	
Male	898 (46.29%)
Female	1042 (53.71%)
Age (Yr)	
<18	58 (2.99%)
18–65	1682 (86.70%)
>65	200 (10.31%)
Courses of disease (Yr)	
<2	64 (3.30%)
2–5	176 (9.07%)
5–10	245 (12.63%)
>10	283 (14.59%)
Unknown	1172 (60.41%)
Health insurance	
Public	1285 (66.24%)
Self-paying	655 (33.76%)

### 3.2 Total antipsychotic prescription among outpatients

A total of 1470 (75.77%) were prescribed antipsychotic medications while the rest of the patients (n = 470, 24.23%) were not prescribed any antipsychotic medication. Among the patients prescribed antipsychotic medications, the majority (83.53% n = 1228) patients were prescribed second-generation antipsychotics (SGAs), while 202 patients were treated only with typical antipsychotics, and 40 were prescribed both FGAs and SGAs. In terms of the treatment approaches of patients, 1238 (84.22%) patients were receiving antipsychotic monotherapy, while 232 (15.78%) patients were prescribed with 2 or more antipsychotics.

### 3.3 Co-prescribing with other psychotropic classes

Most patients with schizophrenia, who were treated with antipsychotic medicaments, were also co-treated with other classes of psychotropic medications. Table 2 outlines the use of antipsychotic combinations in the total antipsychotic prescriptions in the study. For all subjects, intermediate-acting sedative benzodiazepines were the most commonly co-prescribed psychotropic class at 23.66%. Meanwhile, the most frequently prescribed antidepressant was selective serotonin reuptake inhibitors, accounting for 9.79% of the total number. Moreover, mood stabilizers, as one of the neuropsychiatric co-medications, and antihistamines were also generally co-prescribed to patients.

**Table 2:** Percentage of antipsychotic users with other psychotropic drugs.

Concomitant medications	Drug classes^∗^	N = 1940
Benzodiazepines	Intermediate-acting drug (alprazolam, estazolam, oxazepam, lorazepam, nitrazepam)	459 (23.66%)
	Long-acting drug (diazepam, clonazepam)	48 (2.32%)
Antidepressant	SSRIs (fluoxetine, paroxetine, citalopram, escitalopram, fluvoxamine, sertraline)	190 (9.79%)
	SNRIs (venlafaxine, duloxetine, milnacipran)	40 (2.06%)
	SARIs (trazodone)	37 (1.91%)
	NassAs (mirtazapine)	12 (0.62%)
	TCAs (amitriptyline, doxepin, clomipramine)	12 (0.62%)
	TeCAs (maprotiline)	3 (0.15%)
	Others (agomelatine, flupentixol and melitracen tablets, voloxetine)	13 (0.67%)
Mood stabilizer (including antiepileptics)	Sodium valproate, lamotrigine, carbamazepine and lithium carbonate	121 (6.24%)
Antihistamine	Promethazine	38 (1.96%)

### 3.4 Antipsychotic prescriptions from different courses of the disease

Among the 1940 patients conducted in this study, 768 (39.59%) patients could be traced back to the progress note, but only 563 (29.02%) patients had prescription antipsychotics and progress notes meanwhile during the visit. The use of FGAs and SGAs in each course of patients was shown in Table 3, in which the proportion of single SGAs prescriptions was significantly higher than that of single FGAs antipsychotics in each course of monotherapy group, especially among patients with the course less than 2 years (96.08%) and 2 to 5 years (95.65%).

**Table 3:** Patterns of prescribing antipsychotics to patients with varies courses of schizophrenia (n = 563)^∗^.

Course of disease (Year)	Monotherapy of FGAs	Monotherapy of SGAs	Polypharmacy of SGAs and FGAs
<2 (n = 51)	2 (3.92%)	49 (96.08%)	0
2–5 (n = 138)	4 (2.90%)	132 (95.65%)	1 (0.72%)
5–10 (n = 186)	8 (4.30%)	(94.09%)	3 (1.61%)
>10 (n = 189)	26 (13.76%)	154 (81.48%)	9 (4.76%)

The prescriptions of SAGs to patients were presented in Table 4 in order to provide a clear vision of the use of medications in terms of various courses of schizophrenia. In terms of total prescribed antipsychotic medication, risperidone (n = 155) was most frequently prescribed during the study. In addition, aripiprazole (n = 124) and amisulpride (n = 118) were the most common second and third medications among patients prescribed with SGAs. Obviously, patients with a long course of schizophrenia (>10 years) were more often prescribed with risperidone (n = 64). However, no statistical significant was found in comparison with other medications. The loss of progress notes with these subjects (loss to follow-up bias) and systematic errors were the possible causes.

**Table 4:** The percentage of prescriptions of second-generation antipsychotics to patients with varies courses of schizophrenia (n = 523)^∗^.

Course of disease	Risperidone	Paliperidone	Aripiprazole	Olanzapine	Amisulpride	Quetiapine	Clozapine	Ziprasidone	Blonanserin
(Year)	(n = 155)	(n = 79)	(n = 124)	(n = 104)	(n = 118)	(n = 26)	(n = 3)	(n = 16)	(n = 7)
<2	12 (7.74%)	4 (5.06%)	10 (8.06%)	12 (11.54%)	13 (11.02%)	3 (11.54%)	0	0	1 (14.29%)
2–5	37 (23.8%)	12 (15.19%)	35 (28.23%)	22 (21.15%)	38 (32.20%)	8 (30.77%)	1 (33.33%)	4 (25.00%)	2 (28.57%)
5–10	42 (27.10%)	40 (50.63%)	47 (37.90%)	36 (34.62%)	31 (26.27%)	3 (11.54%)	0	9 (56.25%)	1 (14.29%)
>10	64 (41.29%)	23 (29.11%)	32 (25.80%)	34 (32.70%)	36 (30.51%)	12 (46.15%)	2 (66.67%)	3 (18.75%)	3 (42.86%)

### 3.5 Age patterns of antipsychotic use

Patients were stratified by age in Table 5: younger than 18 years (58 cases, 2.99%), 18 to 65 years (1,682 cases, 86.70%), and older than 65 years (200 cases, 10.31%). For the younger patients who were less than 18 years, the highest prescribe rate of antipsychotics is amisulpride (n = 17, 9.29%) But for the patients among 18 to 65 years, the most frequently used antipsychotic was risperidone (n = 363, 82.69%).

**Table 5:** The percentage of prescriptions with second-generation antipsychotics use among different age groups (n = 1940)^∗^.

Age	Risperidone	Aripiprazole	Amisulpride	Olanzapine	Paliperidone	Quetiapine	Ziprasidone	Blonanserin	Clozapine
(Year)	(n = 439)	(n = 270)	(n = 183)	(n = 263)	(n = 165)	(n = 103)	(n = 17)	(n = 7)	(n = 2)
<18 (n = 58)	17 (3.87%)	9 (3.33%)	17 (9.29%)	13 (4.94%)	4 (2.42%)	1 (0.97%)	0	0	0
18–65 (n = 1682)	363 (82.69%)	254 (94.07%)	164 (90.16%)	226 (85.93%)	155 (93.94%)	82 (79.61%)	16 (94.12%)	7 (100.00%)	2 (100.00%)
>65 (n = 200)	59 (13.44%)	7 (4.12%)	2 (1.09%)	24 (9.13%)	6 (3.64%)	20 (19.42%)	1 (5.88%)	0	0

### 3.6 Initiated on long-acting injectable antipsychotics

The study included 99 patients initiated on LAIs. Five kinds of injections could be prescribed in Beijing Anding Hospital: SGA LAI agents, as paliperidone palmitate injection, risperidone microsphere injection, and FGA LAIs (haloperidol injection, haloperidol anilic acid injection and perphenazine injection). Nearly half of the patients (43 cases, 43.43%) were covered on the SGA LAIs therapy during the study period.

### 3.7 Characteristics associated with antipsychotic polypharmacy

The factors related to polypharmacy were shown in Table 6, 563 subjects (29.02%) with valid information on the clinical course were analyzed by multivariate logistic regression. It found that those who prescribed with antipsychotic polypharmacy were characterized by a significant longer course of the disease, higher prescribing rates of LAIs and higher self-paying cost.

**Table 6:** Characteristics associated with antipsychotic polypharmacy.

	OR	95% CI	*P*
Age	**0.977**	0.952–1.001	.064
Disease course	1.049	1.014–1.087	.006
Self-paying cost	2.511	1.506–4.186	<.001
SGAs	4.393	0.962–20.063	.056
LAI antipsychotic use	3.073	1.330–7.099	.009

## 4 Discussion

In this real-world cross-sectional observational study, the patterns of prescribing antipsychotics in 1940 schizophrenia patients from one of the largest tertiary psychiatric hospitals in China were presented. The overall and different populations of antipsychotic use and concomitant use of other psychiatric medications were considered in the study. The main findings of this study were characterized by higher prescription rates of second-generation antipsychotics (83.53%) in the total outpatient's prescriptions. Of total antipsychotic prescriptions in this study, risperidone was the most frequently prescribed, followed by aripiprazole, amisulpride, and olanzapine. LAIs gradually started to be prescribed and were associated with improved adherence. More than 5 times of patients received antipsychotic monotherapy (84.22%) compared with polypharmacy (15.78%).

Compared with the higher prescribing rate of SGAs (63.30%) from this study, the Research on Asia Psychotropic Prescription (REAP) studies^[11]^ revealed the under-prescription of SGAs in East Asian countries and regions including China, Singapore, Korea, Japan, and Taiwan (China). Since the 2000 s, SGAs have been recommended as first-line treatment for schizophrenia in clinical practice guidelines.^[7]^ Therefore, the widespread use of SGAs in this study may be affected by the implementation of clinical guidelines, socioeconomic factors, public health policy, as well as treatment concepts from psychiatrists in Beijing. The higher rate of SGAs prescription was also found in patients with shorter courses of the disease by comparison with outpatients with longer courses. The choice of SGAs, such as paliperidone, aripiprazole, and amisulpride, indicated that psychiatrists and families pay more attention to the treatment and quality of life outcomes of patients, not only functional recovery but also expectations on less effect on metabolism and hyperprolactinemia.^[12,13]^ On the other hand, approximately 10% of patients were prescribed first-generation antipsychotics in this study, especially for older patients with a longer course of the disease, which may be due to the lower price and local clinical traditions of FGAs contributed to its popularity.^[14]^

The prescribing rate of antipsychotic is similar as LANCET's latest published network meta-analysis for 32 oral antipsychotics showed,^[15]^ the highest prescribing rate of risperidone in this study was as the same as reported by REAP studies and Gaviria, Franco et al, 2015.^[16]^ The possible reasons might be:

1. as a SGA, risperidone has superior efficacy and effectiveness with less side-effect;^[15]^
2. with the diversity of its formulations (long-acting injections and multiple oral preparations), which could meet different treatment needs of patients;
3. its main activity metabolite, paliperidone, has more diverse dosage forms, for instance, long-acting injections with a monthly injections and a three-monthly injection are available on the market.

Olanzapine is the most effective medication in terms of discontinuation rates,^[17]^ however, it has a large burden on metabolism presented by gaining greater weight.^[18]^ The latest American Psychiatric Association practice guideline does not recommend olanzapine as the first-line medications.^[19]^ Clozapine is one of the cheapest SGAs in China, which is widely affordable for all patients in China, especially from rural areas. However, with the severe side effect of Clozapine, a lower prescribing proportion of Clozapine is prescribed in the study, which showed a huge difference from an Asian study with the prescribing rate in 14.5% to 15.9% during 2001 to 2009.^[20]^ The decreasing prescription of clozapine was contributed by the efforts of the strict implementation of treatment guidelines and the widespread availability of other SGAs. Furthermore, several SGAs are approved worldwide in recent years for adolescents to treat schizophrenia including risperidone, paliperidone, aripiprazole, olanzapine, lurasidone, and quetiapine, while only paliperidone and aripiprazole are approved in China for childhood-onset schizophrenia. However, amisulpride is the most commonly used in clinical practice among adolescent patients in the study. Therefore, there is an urgent need for more medications or quicker approvals for new drugs to the market for adolescent patients, especially in China.

Even if there are studies show that treatment interruptions with oral medications are by far one of the main causes of schizophrenia relapse and rehospitalization,^[21]^ the use of LAIs should be considered in optimize adherence, improve treatment outcomes, and enhance functioning by reducing the frequency of administration,^[22,23]^ the prescribing rate of long-acting injectable formulations of risperidone and paliperidone in this study isvery low. The barriers to LAIs use may be insufficient psychiatrists’ clinical understanding of the use of LAIs, the lack of patient insight into the disease and the treatment, and the limitations on healthcare policy.

The rate of the patients who received antipsychotic polypharmacy is lower than the frequency of antipsychotic combinations (42.6%) reported in REAP studies^[11]^ which is across 15 Asian countries and regions, and even lower than the worldwide median of 19.6%.^[26]^ This indicates that the principle of monotherapy for schizophrenia mentioned in the guidelines from different countries and regions^[24,25]^ is well implemented in China. But the polypharmacy regimen in individualized treatment is currently still hard to avoid. The possible reasons are as follows: first, the effectiveness of antipsychotic polypharmacy for patients with refractory schizophrenia. Second, the possibility of cross-tapering was common due to unsatisfactory treatment outcomes in the clinic. It was found in this study that the rate of polypharmacy for self-paying patients was 1.406 times that of outpatients with public medical insurance. Because most of the self-paying subjects come from other provinces in China, they had been treated in the local hospitals with ineffective treatment outcomes before the visit to this large tertiary psychiatric hospital. Thirdly, low-dose aripiprazole was used as an adjunctive treatment for antipsychotic-induced higher prolactin levels.^[27]^ The frequency of the antipsychotic combination therapy, to some extent, was elevated for this reason. In the meantime, the use of concomitant psychiatric medications was also quite high among outpatients in this study. We should pay closer attention to the possibility of co-occurring substance abuse disorders, especially with the use of benzodiazepine and/or antidepressants.

The study revealed not only the general prescribing information, such as patient population and groups of interest, but also provided prescribing data by various formulations and the comparison of data in other countries and regions. However, there are some study limitations. First, although we obtained a large number of prescribing data, due to the high flow of outpatients and incompatible EMR systems among hospitals, it was tough to trace back the total socio-demographic and clinical characteristics from all subjects, which caused data missing. Second, the survey, as a single center study, did not include all Beijing's psychiatric hospitals. Further studies could collect long-term tracing data with subjects and promote evidence sharing between multiple clinics. Data on adherence patterns, especially different adherence behaviors between the treatment of oral antipsychotics and LAIs, was not assessed as a limitation. Fourth, the associations between the severity of psychotic symptoms (acute versus chronic) and the patterns of antipsychotic prescription need to be further discussed. Moreover, further analyses of the balancing treatment between patients’ characteristics (age and/or course of disease) and complex dosing regimens can be conducted.

## 5 Conclusions

The current status of antipsychotic outpatient prescriptions was reasonable in this representative large tertiary psychiatric hospital. Second-generation antipsychotics showed domination in prescriptions. The use of antipsychotics in patients with different courses had its own characteristics. More concerns should be paid with concomitant psychiatric medications in clinics.

## Author contributions

**Data curation:** Hongye Yu.

**Formal analysis:** Hongye Yu.

**Investigation:** Xiaobing Guo.

**Resources:** Xiaobing Guo.

**Software:** Xiaobing Guo, Hu Wang.

**Writing – original draft:** Xiaobing Guo.

**Writing – review & editing:** Xiaobing Guo.

## References

[1] Jablensky A. The diagnostic concept of schizophrenia: its history, evolution, and future prospects. Dialogues Clin Neurosci 2010;12:271–87. 20954425 10.31887/DCNS.2010.12.3/ajablensky PMC3181977

[2] Charlson FJ Ferrari AJ Santomauro DF. Global epidemiology and burden of schizophrenia: findings from the Global Burden of Disease Study 2016. Schizophr Bull 2018;44:1195–203. 29762765 10.1093/schbul/sby058 PMC6192504

[3] van Os J Kapur S. Schizophrenia. Lancet 2009;374:635–45. 19700006 10.1016/S0140-6736(09)60995-8

[4] Huang Y Wang Y Wang H. Prevalence of mental disorders in China: a cross-sectional epidemiological study. Lancet Psychiatry 2019;6:211–24. 30792114 10.1016/S2215-0366(18)30511-X

[5] Buchanan RW Kreyenbuhl J Kelly DL. The 2009 schizophrenia PORT psychopharmacological treatment recommendations and summary statements. Schizophr Bull 2010;36:71–93. 19955390 10.1093/schbul/sbp116 PMC2800144

[6] Li P Snyder GL Vanover KE. Dopamine targeting drugs for the treatment of schizophrenia: past, present and future. Curr Top Med Chem 2016;16:3385–403. 27291902 10.2174/1568026616666160608084834 PMC5112764

[7] In: National Collaborating Centre for Mental Health (UK). Schizophrenia: Core Interventions in the Treatment and Management of Schizophrenia in Primary and Secondary Care (Update) [Internet]. Leicester (UK): British Psychological Society; March 2009. 20704054

[8] Leucht S. Psychiatric treatment guidelines: doctors’ non-compliance or insufficient evidence? Acta Psychiatr Scand 2007;115:417–9. 17498152 10.1111/j.1600-0447.2007.01030.x

[9] Cai S Lu H Bai Z Wu R Zhao J. Paliperidone extended-release tablets in Chinese patients with schizophrenia: meta-analysis of randomized controlled trials. Neuropsychiatr Dis Treat 2015;11:1817–34. 26229477 10.2147/NDT.S84833 PMC4517523

[10] Li Q Xiang YT Su YA. Antipsychotic polypharmacy in schizophrenia patients in China and its association with treatment satisfaction and quality of life: findings of the third national survey on use of psychotropic medications in China. Aust N Z J Psychiatry 2015;49:129–36. 24923760 10.1177/0004867414536931

[11] Chong MY Tan CH Fujii S. Antipsychotic drug prescription for schizophrenia in East Asia: rationale for change. Psychiatry Clin Neurosci 2004;58:61–7. 14678459 10.1111/j.1440-1819.2004.01194.x

[12] Weinbrenner S Assion HJ Stargardt T Busse R Juckel G Gericke CA. Drug prescription patterns in schizophrenia outpatients: analysis of data from a German health insurance fund. Pharmacopsychiatry 2009;42:66–71. 19308881 10.1055/s-0028-1103293

[13] Montalvo I Ortega L López X. Changes in prolactin levels and sexual function in young psychotic patients after switching from long-acting injectable risperidone to paliperidone palmitate. Int Clin Psychopharmacol 2013;28:46–9. 23232756 10.1097/YIC.0b013e32835ba832

[14] Correll CU Shaikh L Gallego JA. Antipsychotic polypharmacy: a survey study of prescriber attitudes, knowledge and behavior. Schizophr Res 2011;131:58–62. 21419603 10.1016/j.schres.2011.02.016 PMC3159758

[15] Huhn M Nikolakopoulou A Schneider-Thoma J. Comparative efficacy and tolerability of 32 oral antipsychotics for the acute treatment of adults with multi-episode schizophrenia: a systematic review and network meta-analysis. Lancet 2019;394:939–51. 31303314 10.1016/S0140-6736(19)31135-3 PMC6891890

[16] Gaviria AM Franco JG Aguado V. A non-interventional naturalistic study of the prescription patterns of antipsychotics in patients with Schizophrenia from the Spanish Province of Tarragona. PLoS One 2015;10:e0139403. 26427051 10.1371/journal.pone.0139403 PMC4591292

[17] de Araujo AN de Sena EP de Oliveira IR Juruena MF. Antipsychotic agents: efficacy and safety in schizophrenia. Drug Healthc Patient Saf 2012;4:173–80. 23236256 10.2147/DHPS.S37429 PMC3516452

[18] Leucht S Cipriani A Spineli L. Comparative efficacy and tolerability of 15 antipsychotic drugs in schizophrenia: a multiple-treatments meta-analysis. Lancet 2013;382:951–62. 23810019 10.1016/S0140-6736(13)60733-3

[19] In: 2019 exceptional surveillance of psychosis and schizophrenia in adults: prevention and management (NICE guideline CG178) [Internet]. London: National Institute for Health and Care Excellence (UK); March 12, 2019. 31869042

[20] Xiang YT Ungvari GS Correll CU Chiu HF Shinfuku N. Trends in the access to and the use of antipsychotic medications and psychotropic co-treatments in Asian patients with schizophrenia. Epidemiol Psychiatr Sci 2016;25:09–17. 10.1017/S2045796015000694 PMC6998674 26289066

[21] Song X El Khoury AC Brouillette M Smith D Joshi K. Treatment discontinuation of long-acting injectables or oral atypical antipsychotics among Medicaid recipients with schizophrenia. J Med Econ 2019;22:1105–12. 31062998 10.1080/13696998.2019.1615927

[22] Pilon D Joshi K Tandon N. Treatment patterns in Medicaid patients with schizophrenia initiated on a first- or second-generation long-acting injectable versus oral antipsychotic. Patient Prefer Adherence 2017;11:619–29. 28356723 10.2147/PPA.S127623 PMC5367457

[23] Lafeuille MH Dean J Carter V. Systematic review of long-acting injectables versus oral atypical antipsychotics on hospitalization in schizophrenia. Curr Med Res Opin 2014;30:1643–55. 24730586 10.1185/03007995.2014.915211

[24] Miller AL Chiles JA Chiles JK Crismon ML Rush AJ Shon SP. The texas medication algorithm project (TMAP) schizophrenia algorithms. J Clin Psychiatry 1999;60:649–57. 10549680 10.4088/jcp.v60n1002

[25] Taylor DM Young C Paton C. Prior antipsychotic prescribing in patients currently receiving clozapine: a case note review. J Clin Psychiatry 2003;64:30–4. 10.4088/jcp.v64n0107 12590620

[26] Gallego JA Bonetti J Zhang J Kane JM Correll CU. Prevalence and correlates of antipsychotic polypharmacy: a systematic review and meta-regression of global and regional trends from the 1970 s to. Schizophr Res 2012;138:18–28. 22534420 10.1016/j.schres.2012.03.018 PMC3382997

[27] Meng M Li W Zhang S. Using aripiprazole to reduce antipsychotic-induced hyperprolactinemia: meta-analysis of currently available randomized controlled trials. Shanghai Arch Psychiatry 2015;27:04–17. 10.11919/j.issn.1002-0829.215014 PMC4372756 25852251

